# A Longer Quarantine Period May Be Needed for Effective Control of COVID-19 Transmission: Experience From Odisha, India

**DOI:** 10.7759/cureus.24999

**Published:** 2022-05-14

**Authors:** Manoja K Das, Subham R Nayak, Ashoka Mahapatra, Kishore K Behera, Vinay Kumar Hallur

**Affiliations:** 1 Public Health, The INCLEN Trust International, New Delhi, IND; 2 Microbiology, All India Institute of Medical Sciences, Bhubaneswar, IND; 3 Endocrinology, All India Institute of Medical Sciences, Bhubaneswar, IND

**Keywords:** india, quarantine, incubation period, covid-19, sars-cov-2

## Abstract

Background

The novel coronavirus disease (COVID-19) has become pandemic. For effective disease control, quarantine of the infected and exposed cases for an optimal period is critical. Currently, infected individuals are quarantined for 14 days. We tried to check if the quarantine period practiced is optimal in the Indian context.

Methods

This cross-sectional study was conducted in Odisha, India. We compiled and analyzed the information of 152 laboratory-confirmed SARS-CoV-2 positive cases. Descriptive analysis was conducted.

Results

Out of the 152 cases, 80% were males, 9.8% were symptomatic, 66.4% had travel history, and 53.9% had contact with COVID-19 cases. The incubation period ranged from 1-50 days with a median of 19.5 days (IQR: 17-27 days). The median periods were similar according to gender, history of contact, and presence of symptoms. Interestingly, 84.7% of the cases had an incubation period of more than 14 days. To cover 95% and 90% of the individuals, the quarantine period may have to be extended to 38 days and 35 days, respectively.

Conclusion

A longer observed incubation period (minimum 28 days) suggests the extension of the quarantine period for adults beyond the presently practiced 14 days. Considering the fast-spreading outbreak, an extended quarantine period for 28 days or active periodic follow-up could be more effective.

## Introduction

The novel severe acute respiratory syndrome coronavirus 2 (SARS-CoV-2) pandemic, the deadliest in the century, has spread to 220 countries. The coronavirus 2019 (COVID-19) has infected over 516 million and killed over 6.3 million (1.2%) people globally (as of May 2, 2022). In India, out of the 43.1 million COVID-19 infected individuals, 523,975 (1.1%) have died (as of May 5, 2022). Although individuals of all ages have been infected, lesser symptomatic infections in children and higher fatalities in the elderly (>60 years of age) and those with underlying comorbidities have been observed [1,2]. Lockdown, movement restrictions, and quarantine of infected and suspected individuals have been tried to curtail the SARS-CoV-2 infection transmission, with variable results.

The SARS-CoV-2 infection is transmitted by droplets, aerosols, contaminated surfaces or objects, and oral-fecal modalities [1,3]. The virus can survive on various surfaces from a few hours to days [4]. A higher reproduction (R0) rate for SARS-CoV-2 (2.6-4.71) has been observed compared to other coronaviruses (SARS-CoV, 1.77) [2]. The average incubation period observed was 2-11 days (average 4.8±2.6 days) and an outer limit of 24 days [2,5]. One of the critical steps in controlling SARS-CoV-2 transmission is the quarantine of the infected and potentially exposed individuals. For effective stoppage of transmission, quarantine must be adequate to cover the infectious period. The majority of the incubation periods have been derived from the hospitalized patients and a few community clusters [1,6]. As the majority (80-90%) of the SARS-CoV-2 infected individuals remain asymptomatic or may be pre-symptomatic and don't require hospitalization, there is a need to document the incubation period in symptomatic cases or interval period for reverse transcriptase-polymerase chain reaction (RT-PCR) positivity in asymptomatic cases in community settings. Additionally, it is also important to document whether the incubation period changes in different contexts for devising an appropriate infection control strategy. We wanted to check if the currently practiced 14-day quarantine period for SARS-CoV-2 is optimal in the Indian context. For this study, we used the data on SARS-CoV-2 positive cases available in the public domain from Odisha state, India.

## Materials and methods

This cross-sectional study targeted the SARS-CoV-2 infected cases from Odisha state, India, from March to May 2020. The Indian government has posted the data on SARS-CoV-2 and COVID-19 daily on the dedicated website [7]. Odisha State Government was posting the de-identified information about the SARS-CoV-2 and COVID-19 infected cases daily through press briefings, the portal, and Twitter [8-9]. These contain several details of the patients, including the age, sex, residence, travel history, symptoms, possible exposures, and contact with prior COVID-19 positive cases. We manually extracted and reviewed the data for these SARS-CoV-2 positive/COVID-19 cases from the above source. All these reported SARS-CoV-2/COVID-19 cases were confirmed by the reverse transcription-polymerase chain reaction (RT-PCR) test. Multiple authors extracted the patient data. Two other authors cross-checked the data, and an independent investigator cleaned and coded the data. Information of 188 SARS-CoV-2 positive cases was reported in the portal till May 7, 2020. After that, the data was posted as pooled numbers and not as individual cases. For the SARS-CoV-2 RT-PCR positivity estimation in asymptomatic individuals, we focused on the cases which had exposure dates. Out of the 188 cases, 36 didn't have exposure dates, which were excluded. Thus, the data of 152 cases were analyzed.

The interval between infectious exposure and RT-PCR positivity was considered as the interval for the positive test. For individuals who traveled from outside the state, travel dates to India or Odisha were considered as exposure dates. For the asymptomatic individuals, the dates of infectious exposure and positive SARS-CoV-2 RT-PCR test were used to estimate the interval for the positive test. Continuous data were expressed as proportions, means, and medians with interquartile ranges (IQR). Data between groups according to symptoms (with and without) and contact (with or without) with known SARS-CoV-2 RT-PCR positive tests were compared using the Chi-square test or Mann-Whitney U test. Statistical significance was considered if the p-value was <0.05. Statistical analysis was performed using STATA version 15.0 (Stata Corp LLC, College Station, USA).

The study was approved by the Institutional Ethics Committee at All India Institute of Medical Sciences, Bhubaneswar (Ref No: T/IM-NF/Micro/20/19; May 11, 2020).

## Results

Till May 7, 2020, 188 SARS-CoV-2 positive cases were reported, including 132 active, 54 recovered cases and two deaths. The SARS-CoV-2 RT-PCR positive test rate was 4.2%. Out of the 188 cases, exposure-related information was available for 152 cases to estimate the interval for the positive test. Table 1 reflects the characteristics of the cases. The majority of the cases were males (80%). The overall median age was 40 years (IQR: 29-55 years, range: 3-85 years) and comparable for both genders. Most (85.6%) of the cases were aged 16-60 years. Only 9.8% of the cases were symptomatic. Travel history outside the state was present in 101 (66.4%) cases, including international travel (n=6) and one neighboring state (n=59). A history of contact with suspected or confirmed cases was found in 53.9% of cases. The interval for positive tests ranged from 1 to 50 days, with a median of 19.5 days (IQR: 17-27 days). The median period was similar for the males (22 days, IQR: 17-28 days) and females (18 days, IQR: 16.5-22 days). Only 15.3% of cases had the interval for positive tests ≤14 days. All ten cases with the interval of <7 days had a travel history.

**Table 1:** Characteristics of the SARS-CoV-2 RT-PCR positive cases IQR- Interquartile range; RT-PCR - reverse transcriptase-polymerase chain reaction P-values <0.05 are statistical significant.

Parameters	Pooled (n=152)	History of contact			Presence of symptoms		
		Yes (n=97)	No (n=55)	p-value	Yes (n=15)	No (n=137)	p-value
Age							
Median (IQR) in years	40 (29-55)	40 (29-55)	41 (29-56)	0.168	46 (32-60)	40 (29-55)	0.428
<15 years, %	4	7.1	0	0.12	0	4.4	0.832
16-60 years, %	85.6	81.4	90.9		91.7	85	
≥61 years, %	10.4	11.4	9.1		8.3	10.6	
Demography and exposure parameters							
Males, %	80	70.4	94.5	0	80	78.3	0.064
Symptomatic, %	9.8	11.2	9.1	0.095	-	-	
Contact history, %	53.9	-	-		66.6	63.5	0.095
Travel history, %	66.4	50.5	96.4	0	73.3	65.7	0.153
Quarantined, %	49.1	32.3	70.8	0	28.6	52.1	0.1
Interval for test positivity							
Median (IQR) in days	19.5 (17-27)	19 (17-22)	27 (8-36)	0.802	19.5 (12-24)	19.5 (17-27)	0.505
≤14 days, %	15.3	4.1	35.8	0	26.7	14	0.215
15-24 days, %	56	82.5	7.5		46.7	56.6	
≥25 days, %	28.7	13.4	56.6		26.6	29.4	

Table 1 shows the characteristics of the cases according to the contact/exposure history and presence of symptoms. The majority of the characteristics of these groups were comparable. The median interval for SARS-CoV-2 RT-PCR positive tests was 19.5 days and was similar across the groups except for the cases without any contact/exposure history (27 days, IQR: 8-36 days). Across the groups, the majority of the cases had a positive test interval of >14 days.

## Discussion

Information about the incubation period or interval for test positivity is critical for adopting appropriate intervention measures and determining the quarantine period for preventing SARS-CoV-2 infection transmission. The initial two studies from China informed about the incubation period of SARS-CoV-2 primarily included 100 and 10 hospitalized patients [3,4]. Based on these studies, a quarantine period of 14 days was suggested. Subsequently, systematic reviews and meta-analyses have reported the mean incubation period in the range of 5-7 days [10-12]. Several reports have found that asymptomatic or pre-symptomatic patients can spread SARS-CoV-2 infection [13-17]. Further, in another study, SARS-CoV-2 was shed at higher levels in the upper respiratory tract in pre-symptomatic individuals [18]. These reports question the very premise of the period of quarantine and the cut-off practiced. Hence, in this study, we estimated the interval for a positive test for SARS-CoV-2 cases at pooled and group levels according to the contact exposure and symptom status. The median interval for positive tests was 19.5 days. We observed that 84.7% of the cases had the interval for the positive tests >14 days. The interval for a positive test was also over 21 days when we compared the cases according to the contact history and symptom status. Follow-up data on how many of these asymptomatic individuals became symptomatic was unavailable and hence could not be analyzed further.

Generally, to be effective, a quarantine period should cover incubation periods for about 95% of the cases to be effective [19]. However, since the asymptomatic cases also spread infection, the interval for test-positivity may also be considered instead of the incubation period alone. In this study, a cut-off of 38 days included 95% of the SARS-CoV-2 positive cases (144 of 152). If we assume the median interval for test-positivity of 20 days or 28 days as the cut-off, 53 or 99 additional individuals would have been quarantined, respectively. A study from China, including 9,120 SARS-CoV-2 positive cases, observed the incubation period to be >14 days in 12.5% of cases [20]. A study from Brazil among mildly symptomatic SARS-CoV-2 positive cases documented the average RT-PCR test positivity duration to be 22.67 days (SD: 19.88 days) and 33.34 (SD: 41.71 days) for females and males, respectively [21].

The advised home quarantine in India for individuals having mild or no symptoms for initially 14 days and recently seven days may be insufficient to control the transmission [22,23]. While a longer quarantine period may not be pragmatic, regular follow-up of such patients for another two weeks may be considered.

The potential reasons for this prolonged interval for test positivity are unknown and may be either host-related or virus-related factors. The host immune response, co-morbid conditions, and emerging variants of SARS-CoV-2 could influence the manifestation, severity, and outcome. Whether the virus has undergone some change in its infectivity and host response is to be explored. Further research is needed for COVID-19 infection dynamics and transmissibility in the Indian context, especially in asymptomatic individuals.

The limitations of this study include small sample size, cross-sectional study design, use of the data published on the internet, all the cases were from Odisha state only, and non-availability of data on host-related factors, follow-up, and outcome.

## Conclusions

To conclude, a longer interval for RT-PCR test positivity is being observed in SARS-CoV-2 infected individuals from Odisha, India. Considering the fast-spreading SARS-CoV-2 outbreak, an extension of the quarantine period from 14 days to 28 days along with screening for SARS-CoV-2 by RT-PCR and periodic follow-up of the suspected cases and contacts could reduce virus spread and monitor infection. Also, the infection dynamics and viral shedding period by infected individuals need to be documented to inform the incubation periods appropriately. Further information on the incubation period from other parts of India is required for validation and comparison.
